# A New Mode of Preserving the Ergot of Rye

**Published:** 1844-10-19

**Authors:** R. M. Nunn

**Affiliations:** Wexford


A NEW MODE OF PRESERVING THE ERGOT OF RYE.

BY R. M. NUNN, ESQ., WEXFORD.

Having seen in a late number of the Lancet, (August 10th, page 611) some observations on the preservation of the *secale cornutum*, by Mr. Rawle, of Saffron Walden, and, as it is a subject to which I have given some attention, I can recommend the following plan for the preservation of this valuable but perishable drug as one on which the practitioner can confidently rely. Procure a choice specimen of the ergot, reduce it to powder, have in readiness a sufficient number of two-drachm bottles, into each bottle put one drachm of sulphuric ether (spirits of wine may do as well), and then press in two drachms of the powdered drug (if the bottles are of the proper size it will require a slight pressure to make them hold this quantity); now cork it well, and either seal with wax, or cover with bladder. When required for use, put the contents of one of these bottles into a tumbler, and pour on it a small quantity of boiling water; violent effervescence takes place which quickly subsides, and during which the ether is evaporated; you may then add as much more boiling water as may be necessary. It is *instantly* fit for use.—*Ibid.*

